# Islands and Stepping-Stones: Comparative Population Structure of *Anopheles gambiae sensu stricto* and *Anopheles arabiensis* in Tanzania and Implications for the Spread of Insecticide Resistance

**DOI:** 10.1371/journal.pone.0110910

**Published:** 2014-10-29

**Authors:** Deodatus Maliti, Hilary Ranson, Stephen Magesa, William Kisinza, Juma Mcha, Khamis Haji, Gerald Killeen, David Weetman

**Affiliations:** 1 Ifakara Health Institute, Environmental Health and Ecological Sciences Thematic Group, Ifakara, Morogoro, United Republic of Tanzania; 2 University of Glasgow, Institute of Biodiversity Animal Health and Comparative Medicine, Glasgow, Lancashire, United Kingdom; 3 Department of Vector Biology, Liverpool School of Tropical Medicine, Merseyside, Liverpool, United Kingdom; 4 Zanzibar Malaria Elimination Programme, Unguja, Zanzibar, United Republic of Tanzania; 5 RTI International, Global Health Division, Dar es Salaam, United Republic of Tanzania; 6 National Institute for Medical Research, Amani Research Center, Muheza, Tanga, United Republic of Tanzania; Virginia Tech, United States of America

## Abstract

Population genetic structures of the two major malaria vectors *Anopheles gambiae* s.s. and *An. arabiensis*, differ markedly across Sub-Saharan Africa, which could reflect differences in historical demographies or in contemporary gene flow. Elucidation of the degree and cause of population structure is important for predicting the spread of genetic traits such as insecticide resistance genes or artificially engineered genes. Here the population genetics of *An. gambiae* s.s. and *An. arabiensis* in the central, eastern and island regions of Tanzania were compared. Microsatellite markers were screened in 33 collections of female *An. gambiae* s.l., originating from 22 geographical locations, four of which were sampled in two or three years between 2008 and 2010. *An. gambiae* were sampled from six sites, *An. arabiensis* from 14 sites, and both species from two sites, with an additional colonised insectary sample of each species. Frequencies of the knock-down resistance (*kdr*) alleles 1014S and 1014F were also determined. *An. gambiae* exhibited relatively high genetic differentiation (average pairwise F_ST_ = 0.131), significant even between nearby samples, but without clear geographical patterning. In contrast, *An. arabiensis* exhibited limited differentiation (average F_ST_ = 0.015), but strong isolation-by-distance (Mantel test r = 0.46, p = 0.0008). Most time-series samples of *An. arabiensis* were homogeneous, suggesting general temporal stability of the genetic structure. *An. gambiae* populations from Dar es Salaam and Bagamoyo were found to have high frequencies of *kdr* 1014S (around 70%), with almost 50% homozygote but was at much lower frequency on Unguja Island, with no. *An. gambiae* population genetic differentiation was consistent with an island model of genetic structuring with highly restricted gene flow, contrary to *An. arabiensis* which was consistent with a stepping-stone model of extensive, but geographically-restricted gene flow.

## Introduction

In sub-Saharan Africa, a dramatic increase in household ownership of long-lasting insecticidal nets (LLINs), is considered one of the major factors contributing to the fall in malaria cases over the last decade [1]. Sustainability of LLINs as a frontline control strategy against malaria is threatened by growing *Anopheles* resistance to pyrethroids [2], the only class of insecticides licensed for LLIN treatment. Improved understanding of the mechanisms responsible for insecticide resistance in *Anopheles* malaria vectors, and development of reliable diagnostics (such as those available for *kdr* knockdown resistance mutations [3]) are considered important goals to prolong the efficacy of pyrethroids for mosquito control [4]. A less well-studied aspect of vector control concerns how resistance spreads, though such information is important to permit optimised targeting of interventions using complementary insecticides and insecticide combinations. Genetic data can aid predictions of the spread of resistance alleles via inference of vector population structure, which can be compared to the spatial or temporal distribution of diagnostic markers for specific resistance mechanisms where these are available [5]. In addition, vector population genetic data could potentially give insight into connectivity of disease transmission dynamics [6], and is also an essential prerequisite for rational planning of vector control strategies that focus on release of sterilised or genetically manipulated mosquitoes [7].

Until recently, *An. gambiae* s.s. was considered the principal malaria vector across most parts of Sub-Saharan Africa, but several areas of East Africa have shown a recent frequency shift towards *An. arabiensis*. Though a causal link remains to be demonstrated, this is coincident with scaling-up of LLIN distribution [8]–[10]. Potentially paradoxically, *An. arabiensis* is typically less resistant to pyrethroids than *An. gambiae* s.s. when sympatric populations are compared, and usually shows lower frequencies (often complete absence) of known target site resistance mechanisms, such as the *kdr* 1014 mutations [11]–[14]. However, *An. arabiensis* is considered more adaptable in blood-feeding behaviour in being more zoophagic, exophagic and exophilic [15]–[20]. *An. arabiensis* also exhibits greater resilience to arid conditions [21] than *An. gambiae* s.s. (i.e. S-molecular form; [22]), and appears to avoid dramatic changes in effective population size (*N_e_*) across wet and dry periods, even in extremely seasonal environments [23]. As a consequence, *An. arabiensis* might be predicted to exhibit more widespread homogeneity in population structure than *An. gambiae s.s*.

In West Africa, the marked population structure in *An. gambiae s.s.* tends to be associated with divergence between molecular and chromosomal forms [24]–[26], although the level of differentiation detected can depend upon the type of markers studied (e.g. microsatellites *vs* single nucleotide polymorphisms, SNPs) and/or their genomic location [27]–[29]. The inhospitable environments of the Rift Valley Complex have been identified as a major barrier partitioning East African *An. gambiae s.s.* populations [30], [31] but appear to have relatively little impact on gene flow in *An. arabiensis* [32], [33], perhaps reflecting greater ecological tolerance. In contrast, geographical isolation on islands can cause substantial differentiation and reduced diversity, relative to mainland populations, in both species [34]–[36]. Ecological zonation has also occasionally been associated with strong population structure (F_ST_>0.1; where F_ST_ is a widely applied metric measuring within *vs* among population diversity) in country-wide surveys of *An. gambiae* s.s. (M and S forms) in Ghana [37] and *An. arabiensis* in Nigeria [38]. Moreover, the Nigerian study was one of few in either species to detect significant isolation by distance, although in this case patterns of genetic diversity suggest that historical range expansion [39], may have played a greater role than contemporary geographical restriction of gene flow [38]. At small spatial scales (e.g. <200 km; [40]), and in the absence of variation in molecular or chromosomal forms in West African *An. gambiae* s.s., differentiation is usually low in both species [32], [40]–[42], often falling below the limits of detection of the microsatellite marker panels applied. Therefore studies to date partially support a hypothesis of weaker population structure in *An. arabiensis* than *An. gambiae* s.s., a difference which might be compounded by relative range expansion and contraction in the latter. In contrast, a recent study of comparative population genetic structure in the Kilombero valley of Tanzania reported little differentiation among three *An. gambiae* s.s. samples but strong population structure in *An. arabiensis*, with F_ST_>0.1 at a scale of <100 km, and even suggestion of differentiation within sympatric samples [43].

While *An. gambiae* s.s. populations have recently declined dramatically in many parts of East Africa [8]–[10], [44], [45], this is not consistently the case throughout the region and *An. arabiensis* has proven remarkably persistent despite high coverage of LLINs and, in some areas Indoor Residual Spraying (IRS). While physiological resistance to pyrethroids is now clearly emerging in both species [46], *An. arabiensis* also appears to exhibit a number of behaviours that render it relatively unresponsive to LLINs and IRS, such behaviours include early exit, outdoor resting, outdoor feeding and feeding upon animals [44], [47], [48]. These front line strategies therefore need to be supplemented with complementary vector control measures that improve on the levels of control achieved outdoors [49], [50] and others which target mosquitoes outdoors and/or at source [51]–[53]. Clarification of vector population connectivity within each species in countries like Tanzania, where 73% of the human population live in high malaria transmission areas [4], can aid targeting of interventions and planning of management strategies to combat the spread of insecticide resistance.

Here we present a comparative study of population genetic structure in *An. arabiensis* and *An. gambiae* s.s. across Tanzania to: (1) investigate spatial and temporal population structure; (2) identify possible barriers to gene flow; and (3) determine inter-relationships of (1) and (2) with the frequency of insecticide resistance-associated *kdr* alleles. We report that most *An. gambiae* s.s. samples were differentiated, in some cases strongly, but without clear geographical patterning, consistent with an island model of genetic structure. By contrast, *An. arabiensis* exhibited weak differentiation with strong isolation-by-distance, concordant with a stepping-stone model of extensive, but geographically-restricted gene flow.

## Materials and Methods

### Ethics statement

All mosquitoes were either collected through routine physiological surveillance activities of the National Institute for Medical Research (NIMR) of Tanzania and the Zanzibar National Malaria Elimination Programme (ZAMEP), or through research protocols implemented by the Ifakara Health Institute (IHI) that were approved by both the IHI internal institutional review board (Reference IHI/IRB/A.50) and the Medical Research Coordination Committee at NIMR (Reference NIMR/HQ/R.8a/Vol. IX/801). Informed written consents were obtained from the household owners for permission to perform sampling in and around households.

### Sample sites and species identification

**Figure 1:**
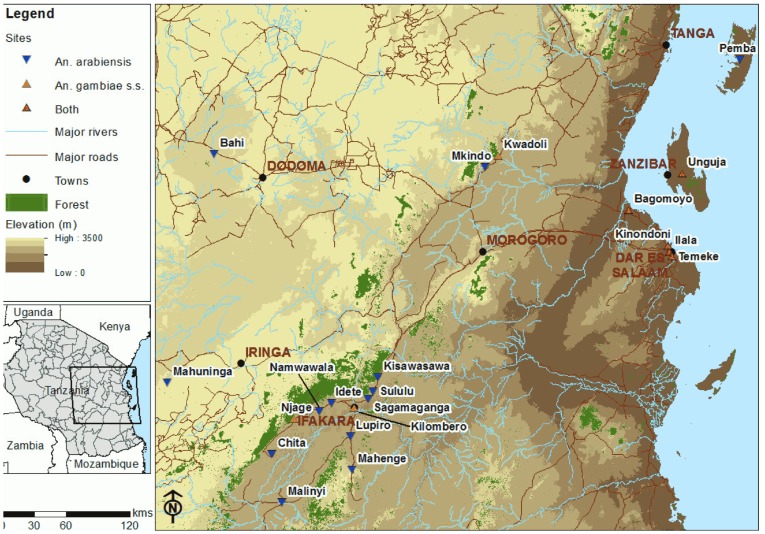
Map of central-eastern Tanzania showing sampling sites.

The study was conducted in three regions of Tanzania (Fig. 1): the south-central area, which includes the highly malaria-endemic Kilombero Valley; the Indian Ocean coast, including three districts of urban Dar es Salaam, and Bagamoyo 60 km to the north; and the Zanzibar islands of Unguja and Pemba. A total of 33 collections, comprising of nine *An. gambiae* s.s. samples and 24 *An. arabiensis* samples were included in the study. Of the 16 collections of *An. arabiensis* from the Kilombero Valley, nine formed a temporal series from the villages of Idete, Namawala and Lupiro sampled between 2008 and 2010. In addition, we included samples from IHI insectary colonies of *An. gambiae* s.s. (colonised in 1996) and *An. arabiensis* (colonised in 2008) as entirely isolated out-groups. A total of 1429 *An. gambiae s.l.* mosquitoes (identified using morphological keys [54]) were collected between 2008 and 2010 using human landing catches (HLC), Centre for Diseases Control (CDC) light traps, Ifakara Tent Traps (ITT), window exit traps, and resting catches inside households using mouth aspirators and back-pack aspirators. All samples were stored dry over silica gel. DNA was extracted from whole *An. gambiae* s.l. using the Livak method [55] and re-suspended in 100 µl of water. Species identity as *An. gambiae* s.s. or *An. arabiensis* was diagnosed using a standard allele-specific PCR method [56] with visualisation of amplicons on a 2% agarose gel.

### Microsatellite and *kdr* genotyping

Twelve microsatellite loci spanning all three chromosomes were genotyped: AGXH678 and AGXH7 from the X chromosome, AG2H79, AG2H786, AG2H799 and 2R_Si_5 from chromosome 2, and AG3H812, AG3H119, AG3H577, AG3H811, AG3H765 and 33C1 from chromosome 3. Primers for loci beginning with the prefix AG were developed by [57], for 33C1 by [58], and for 2R_Si_5 by DW (primers given in [59]). Each locus was amplified in a 15 µl reaction containing 1.5 µl 10X PCR buffer with 1.5 mM MgCl_2_, 0.3 µl 10 mM dNTPs, 0.2 µl 10 mM cy5- or cy5.5-labelled forward primer, 0.15 µl of 10 mM reverse primer, 0.2 µl of Taq, 11.15 µl of PCR-quality water and 1.5 µl of DNA extract. PCR cycles included initial denaturation at 95°C for 5 minutes, followed by 35 cycles of denaturation at 94°C for 30 seconds, annealing at 55°C or 58°C for 1 minute (depending on the optimal annealing temperature of the primers), extension at 72°C for 1 minute and a final extension step of 72°C for 10 minutes. Three pairs of primers with the same annealing temperature, different base pair sizes and different fluorescent labels were amplified in each reaction. PCR products were run on a Beckman-Coulter CEQ 8000 capillary sequencer and sizes scored automatically by comparison with the Beckman-Coulter DNA size standard 400 with all alleles checked manually. *Kdr* L1014F and L1014S genotyping was performed on a randomly-selected sample of 20 individuals from each collection site using TaqMan qPCR [3]. PCR reactions were carried out in 20 µl each containing 10 µl of SensiMix (Bioline), 900 nM of primer, 900 nM of probe, 8.5 µls of PCR quality water and 1 µl of DNA. Samples were run on a Stratagene 3005 (Agilent Technologies) with cycling conditions: 10 minutes at 95°C followed by 40 cycles of 95°C of 10 seconds, and 60°C for 45 seconds.

### Statistical analysis

Microchecker 2.2.3 [60] was used to identify possible scoring errors. Deviation from neutrality of loci was examined using LOSITAN, which uses an F_ST_ outlier approach to detect loci showing extreme variation given their level of polymorphism [61]. Linkage disequilibrium among loci was tested using the exact tests in GENEPOP 4.0 [62], with default settings. Hardy-Weinberg (H-W) equilibrium was tested using FSTAT 1.2 via permutation tests based on the positive or negative magnitude of F_IS_. Genetic diversity was measured by expected heterozygosity (*H_e_*) and allelic richness (*R_s_*) computed by FSTAT 1.2 [63], the latter based on a minimum number of genotypes in any population of nine for *An. gambiae* and 14 for *An. arabiensis*. FSTAT was also used to generate pairwise F_ST_ values between sample sites and to test for population differentiation using the G-test genotypic permutation procedure. Following pooling of temporal samples, isolation-by-distance was examined by comparison of matrices for linearized F_ST_ (F_ST_/1-F_ST_) and the natural logarithm (ln) of geographical distance using a Mantel test with 10 000 permutations implemented by the Poptools add-in for Excel [64]. Insectary samples were excluded from this test. PHYLIP 3.68 [65] was used to produce a neighbour-joining tree from F_ST_ values, again with pooled temporal samples, which was visualised using FIGTREE 1.3 [66]. The Bonferroni procedure was applied throughout to correct for multiple testing. Bayesian clustering analysis of data was performed using two models implemented by BAPS [67]. The first, used for both species, applies a spatially-conditioned non-admixture model to determine clusters of sample sites; an individual-based spatially-conditioned model was also applied to *An. arabiensis*. Both clustering models incorporate geographical location as a prior to aid cluster determination and the individual model works best when individual coordinates are used. Since these were not available we produced them by randomly generated coordinates from within a 10 km radius from the location of each site to assign to individuals based on observations of maximal anopheline flight distances [68]. For the population-level clustering analyses temporal samples from single sites were pooled, however, this does not apply to the individual analysis, which does not use population information as a prior. For all sample collections we attempted estimation of variance effective population sizes via the linkage disequilibrium method implemented in the software LDNE [69], and utilising only alleles with frequency greater than 5%, and tested for mutation-drift equilibrium using the Wilcoxon test in Bottleneck [70], with default settings for each mutation model. Principal component analysis (in SPSS 20) was used to generate a single axis summarising geographical position from latitude and longitude data.

## Results

### Data quality control

Of the microsatellite loci genotyped, only AGH799 proved to be impossible to score reliably and was excluded prior to any analysis. Microchecker highlighted multiple instances of scoring errors, primarily as null alleles, and scoring was checked wherever potential problems were highlighted. Nevertheless, 8 out of 99 tests for H–W disequilibrium in *An. gambiae* s.s. were significant following correction for multiple testing. All but one indicated a deficit of heterozygotes and each was in a different sample, negating the likelihood of within-population structure as an explanation. However, four were significant for locus AGXH7, suggesting the presence of null alleles (Table 1A). Owing to the moderate number of loci available, AGXH7 was retained in the analysis uncorrected but its impact was monitored subsequently. Lositan [61] indicated that locus 33c1 gave a signal of excessive differentiation (Table 1A), and it was removed from subsequent analyses. None of the tests for linkage disequilibrium in *An. gambiae* s.s. were significant following multiple-testing correction, so the loci included were considered to be segregating independently. Data for *An. arabiensis* samples proved more problematic, with 27 tests for H–W disequilibrium significant following correction for multiple testing (Table 1B). Of those indicating a deficit of heterozygotes, all but one involved locus AG3H811, overwhelmingly suggesting null alleles rather than within-population structure as an explanation. Again, AG3H811 was retained in the analysis uncorrected, but its impact was monitored subsequently. Of the 14 significant tests for heterozygote excess, 12 involved loci AG2H78 and AGXH67. These loci, in addition to AG3H765 and 33c1 were identified by Lositan as exhibiting deviations from neutral expectations, thereby unduly influencing estimated differentiation and consequently were excluded. Only one of over 500 tests for linkage disequilibrium was significant following correction for multiple testing, suggesting overall that included loci were segregating independently. Following these quality control procedures, the final dataset was reduced to ten microsatellites for *An. gambiae* s.s. and seven for *An. arabiensis* (Data S1).

**Table 1:** F_IS_ and Lositan values for *An. gambiae s.s*.(A) and *An. arabiensis* (B).

(A) *Anophelesgambiae*																									
		Unguja (2010)	Ilala (2008)	Kinondoni (2008)	Temeke (2008)	Bagamoyo (2008)	Kwadoli (2009)	Kilombero (2009)	Njage (2009)	Insectary (2011)															
Lositan P	F_IS_				F_ST_																				
0.722	AGH765	0.13	0.18	0.22	0.15	0.29	0.07	0.01	0.21	−0.02															
0.494	AG3H811	0.08	0.11	0.06	0.18	0.14	0.14	0.11	0.21	0.07															
0.952	AG2H79	−0.09	−0.01	−0.02	−0.11	−0.17	−0.19	−**0.34**	−0.22	0.11															
0.154	AGXH7	0.42	**0.73**	**0.70**	**0.52**	0.20	**0.52**	0.25	0.12	−0.31															
0.702	AG3H812	0.57	0.13	0.16	0.03	0.18	0.20	0.12	0.11	0.25															
0.750	AG3H119	−0.08	0.22	0.25	0.04	**0.41**	0.14	0.16	0.11	−0.01															
0.790	AG3H577	**0.59**	0.06	0.02	0.02	−0.07	−0.01	0.11	0.08	−0.21															
**0.999**	33C1	−0.02	−0.09	−0.14	0.23	−0.13	0.03	−0.15	−0.01	−0.02															
0.384	2R_Si_5	0.14	0.19	0.24	0.07	0.10	−0.05	0.10	−0.05	−0.09															
0.054	AG2H786	−0.31	0.09	0.01	0.03	0.19	0.12	0.20	−0.02	0.11															
0.952	AGXH678	−0.02	0.08	0.04	−0.01	−0.03	−0.11	0.09	−0.10	**0.80**															

(B)* Anopheles arabiensis*																									
		Pemba (2010)	Unguja (2008)	Unguja (2010)	Dar (2008)	Bagamoyo (2008)	Idete (2008)	Idete (2009)	Idete (2010)	Namawala (2008)	Namawala (2009)	Namawala (2010)	Lupiro (2008)	Lupiro (2009)	Lupiro (2010)	Sululu (2010)	Sawasawa (2010)	Chita (2010)	Malinyi (2010)	Mahenge (2011)	Sagamanganga (2010)	Mkindo (2009)	Mahuninga (2011)	Bahi (2011)	Insectary (2011)
Lositan P	F_IS_											F_ST_													
**1.000**	AGH765	−0.02	−0.09	1.00	0.21	−0.01	−0.05	−0.01	−0.13	−0.30	−0.08	−**0.32**	−0.08	−0.15	0.15	−0.07	−0.01	−0.02	−0.02	−0.04	0.11	0.29	−0.05	0.49	0.45
0.144	AG3H811	**0.42**	**0.65**	0.40	0.08	0.42	−**0.55**	**0.41**	**0.49**	0.21	0.21	0.30	**0.25**	0.21	0.25	0.05	**0.36**	**0.41**	**0.30**	0.28	**0.38**	0.29	**0.42**	**0.37**	**0.40**
0.411	AG2H79	0.11	0.01	0.47	−0.09	−0.24	−0.12	0.20	−0.14	−0.16	0.15	−0.23	0.15	0.19	−0.19	0.21	0.20	0.29	0.23	0.07	0.06	0.03	−0.07	−0.08	0.06
0.043	AGXH7	0.00	0.34	0.20	−0.23	−0.34	−0.11	0.11	−0.14	0.13	−0.03	−0.27	0.16	0.08	−0.17	−0.03	−0.07	−0.07	−0.20	−0.14	−0.06	0.02	0.11	0.13	−0.08
0.001	AG3H812	−0.09	0.15	0.24	−0.02	−0.15	−0.16	−0.01	−0.14	0.01	−0.13	−0.05	0.12	0.16	0.01	−0.23	0.00	0.05	0.09	0.01	0.00	0.01	−0.01	0.06	0.10
0.000	AG3H119	0.05	0.10	0.27	0.00	0.31	0.06	−0.03	−0.14	−0.05	−0.02	−0.05	0.17	0.02	0.22	0.14	−0.04	−0.09	0.01	0.18	−0.07	0.27	0.13	−0.06	0.11
0.001	AG3H577	0.08	0.07	0.42	0.02	0.00	0.21	−0.18	−0.14	−0.14	0.03	−0.04	0.06	0.03	−0.24	−0.09	−0.14	−0.03	−0.07	−0.02	0.18	0.12	0.01	0.02	0.06
**0.993**	33C1	−0.14	−0.12	−0.10	−.08	−0.15	0.07	−0.01	−0.14	0.07	−0.11	−0.17	0.03	−0.08	0.00	0.02	0.08	0.06	0.10	0.04	−.04	0.13	0.01	0.01	0.31
0.080	2R_Si_5	0.02	0.14	−0.13	0.09	−0.10	−0.11	−0.11	−0.14	0.05	0.05	0.15	0.05	0.25	0.03	−0.05	−0.11	0.12	0.07	0.18	0.07	−0.03	0.17	0.02	0.08
**0.995**	AG2H786	0.02	−0.02	−0.07	−0.19	−0.16	−**0.37**	−**0.55**	−0.14	−**0.46**	−0.20	−**0.63**	−0.10	−**0.67**	0.54	0.04	−0.03	0.15	0.07	0.34	0.08	−0.08	0.07	0.19	0.13
**1.000**	AGXH678	0.07	0.05	0.10	−0.07	−0.22	0.10	**0.43**	−0.14	0.38	−**0.65**	−0.12	−**0.41**	0.14	−0.12	−0.04	−**0.61**	−**0.59**	−0.38	−**0.40**	−**0.49**	−0.37	0.01	0.23	0.06

FIS values in bold are significantly greater or larger than expected (following Bonferroni correction).

Lositan P values in red indicate loci showing patterns of differentiation exceeding neutral expectations; these loci were excluded.

### Genetic diversity and differentiation

**Figure 2:**
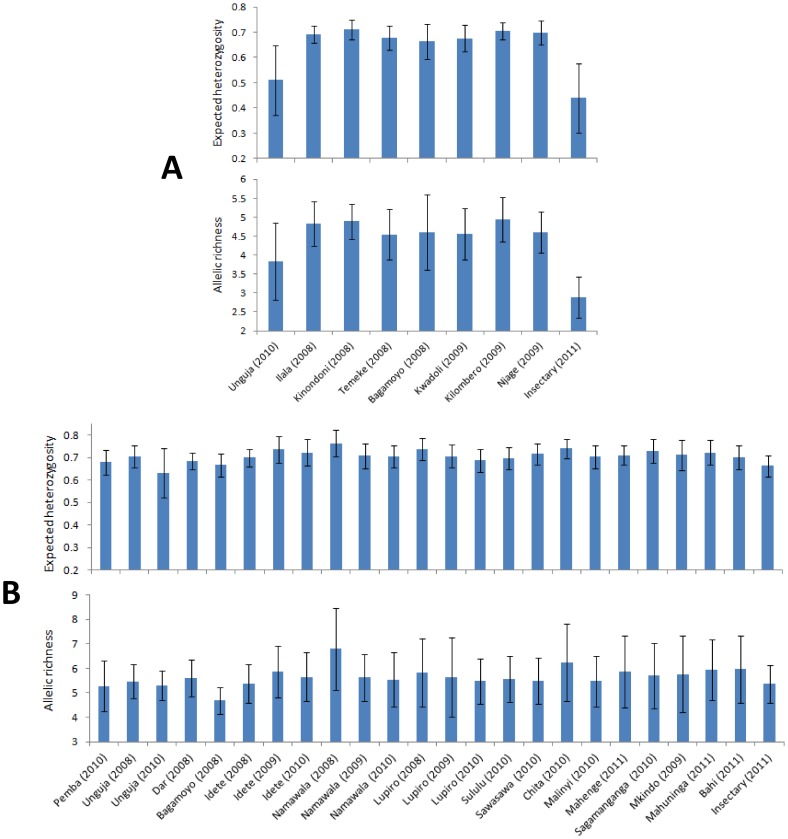
Genetic diversity in (A) *An. gambiae s.s.* and (B) *An. arabiensis*. Each plot shows the mean expected heterozygosity or allelic richness across loci with 95% confidence intervals.

**Figure 3:**
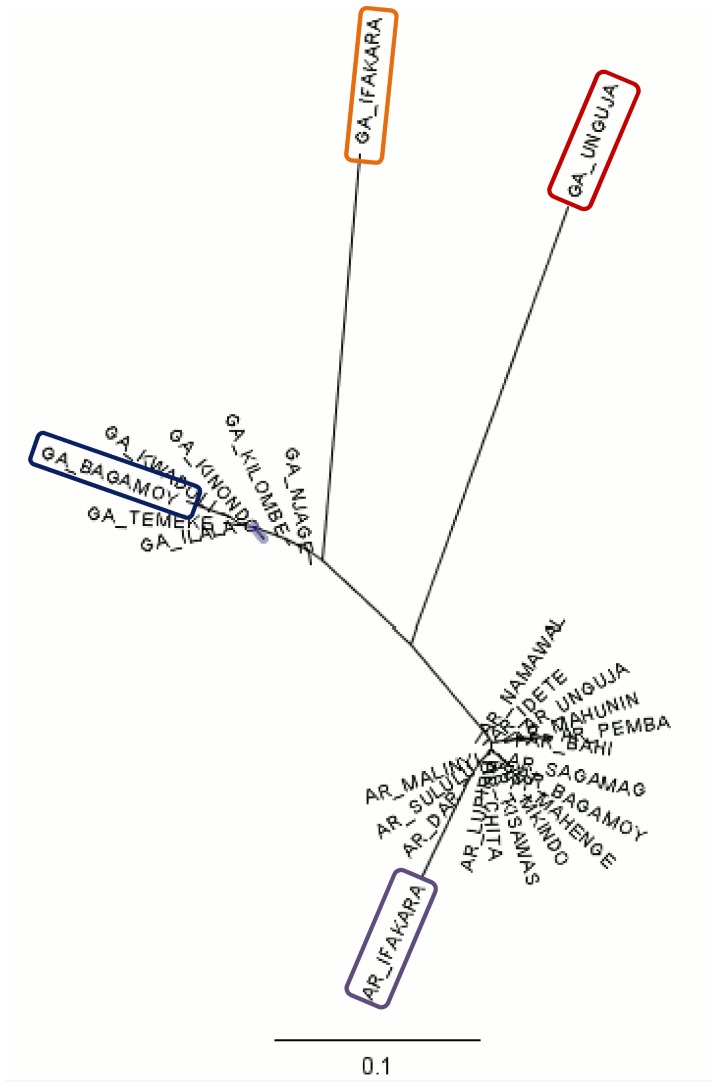
Neighbour-joining tree based on linearised F_ST_. Sample names with the prefix GA are *An. gambiae s.s.*, and those with the prefix AR are *An. arabiensis*. Samples labelled IFAKARA are from insectary colonies. Within each species, samples identified as distinct clusters by BAPS are circled; others fall within a single cluster in each species.

**Figure 4:**
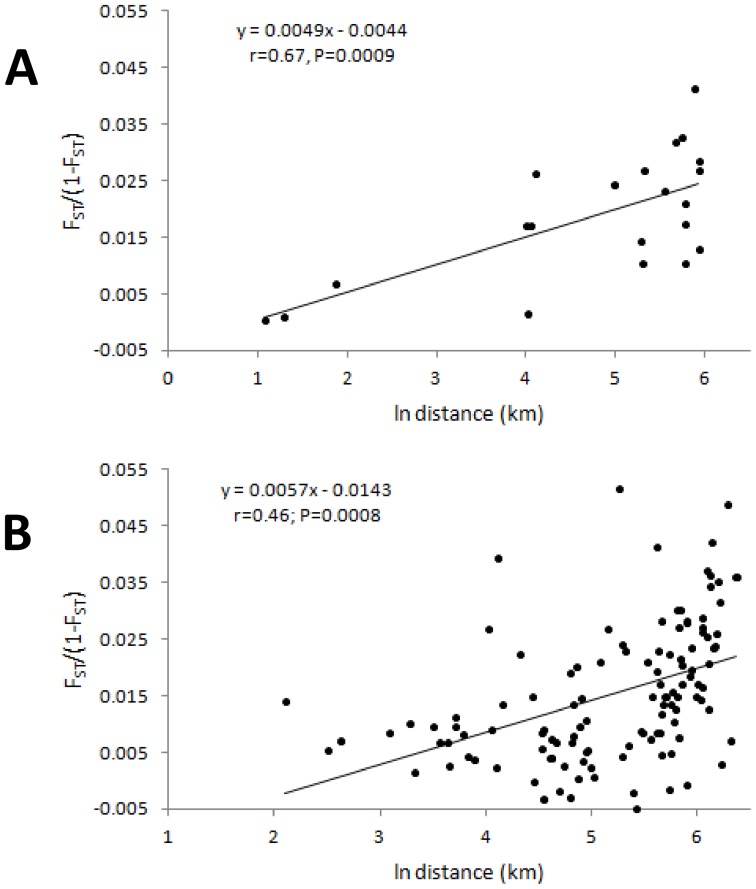
Isolation by distance (IBD) plots in *An. gambiae s.s.* (A, B) and *An. arabiensis* (C). Each plot shows linearised pairwise F_ST_ against the natural logarithm of pairwise distance between sample sites. In (A) all *An. gambiae* sites are included, and the massive differentiation of Unguja (pairwise point in dashed circle) from all other sites obscures the IBD relationship visible in (B) once pairwise comparisons involving Unguja were excluded. In all plots insectary samples are excluded and in (C) temporal samples from the same location were pooled for the analysis. P-values are from Mantel tests.

Whether measured by expected heterozygosity (*H_e_*) or allelic richness (*R_s_*), diversity was generally lower in *An. gambiae* s.s. samples from the island of Unguja and, as expected, the Ifakara insectary (Fig. 2A). In contrast, genetic diversity varied little across *An. arabiensis* samples (Fig. 2B), with even the Ifakara insectary sample exhibiting levels of *H_e_* and *R_s_* comparable to wild populations. *An. gambiae* s.s. exhibited generally moderate population differentiation but most pairwise tests of differentiation were significant (Table 2A). This was highlighted both by F_ST_ levels and by BAPS group-level cluster analysis (which tends to detect higher-level structure) which partitioned Unguja, the insectary sample, and also Bagamoyo as each being distinct from the other *An. gambiae* s.s. samples (Fig. 3). AGXH7, for which null allele(s) were suspected, did not show especially high or low differentiation and its exclusion had no effect on BAPS results. When all data were included, there was no relationship between genetic differentiation and geographic distance (Mantel test r = −0.05, p = 0.38) in *An. gambiae* s.s (Fig. 4A). Following exclusion of the sample from Unguja, for which all pairwise F_ST_ values were much higher than others (Table 2A), a highly significant relationship between genetic differentiation and distance was detectable for *An. gambiae* s.s. (Fig. 4B). In *An. arabiensis*, the majority of pairwise comparisons were not significant, and most F_ST_ values were low to moderate (Table 2B), with the exception of comparisons involving the insectary sample, which was the only one to partition separately in cluster analysis. There was no significant differentiation among the time-series samples of *An. arabiensis* from Unguja, Lupiro or Namawala, nor between Idete samples from 2009 and 2010, though the Idete sample from 2008 was significantly different from 2009 and 2010 (Table 2B).

**Table 2:** (A) *Anopheles gambiae* pairwise F_ST_ values.

Sample	N	Latitude	Longitude	PC1	1	2	3	4	5	6	7	8	9															
Unguja (2010)	31	−6.171	39.314	0.912		*	*	*	*	*	*	*	*															
Ilala (2008)	48	−.816	39.266	0.473	0.319		NS	NS	*	*	*	*	*															
Kinondoni (2008)	39	−6.792	39.255	0.484	0.306	0.000		*	*	*	*	*	*															
Temeke (2008)	49	−6.841	39.287	0.465	0.322	0.001	0.007		*	*	*	*	*															
Bagamoyo (2008)	46	−6.453	38.897	0.556	0.327	0.017	0.017	0.026		*	*	*	*															
Kwadoli (2009)	46	−6.053	37.634	0.293	0.328	0.010	0.014	0.026	0.024		*	*	*															
Kilombero (2009)^k^	45	−8.140	36.681	−1.460	0.314	0.010	0.017	0.021	0.032	0.023		NS	*															
Njage (2009)^k^	43	−8.230	36.190	−1.722	0.324	0.013	0.028	0.026	0.040	0.031	0.002		*															
Insectary (2011)^k^	51	n/a	n/a	n/a	0.485	0.250	0.268	0.273	0.281	0.247	0.229	0.223																

(B) *Anopheles arabiensis*																												
Sample	N	Latitude	Longitude	PC1	1	2	3	4	5	6	7	8	9	10	11	12	13	14	15	16	17	18	19	20	21	22	23	24
Pemba (2010)	46	−5.179	39.763	2.380		NS	*	*	*	*	*	*	*	*	*	*	*	*	*	*	*	*	*	*	*	*	NS	*
Unguja (2008)	51	−6.171	39.314	1.678	0.016		NS	NS	*	*	NS	NS	NS	NS	NS	*	NS	NS	*	*	NS	*	*	*	NS	NS	NS	*
Unguja (2010)	42	−6.171	39.314	1.678	0.039	0.014		NS	NS	*	*	NS	*	NS	NS	*	NS	NS	NS	*	NS	NS	NS	*	NS	*	NS	*
Dar (2008)	31	−6.841	39.287	1.320	0.049	0.024	0.034		*	*	*	NS	*	*	*	*	*	NS	NS	*	*	*	*	*	NS	*	*	*
Bagamoyo (2008)	24	−6.453	38.897	1.356	0.026	0.031	0.032	0.038		*	NS	NS	*	NS	NS	NS	NS	NS	NS	NS	NS	NS	NS	NS	NS	NS	NS	*
Idete (2008)^k^	37	−8.099	36.484	−0.514	0.060	0.051	0.083	0.054	0.020		*	*	*	*	*	*	*	*	NS	*	*	*	*	*	*	*	*	*
Idete (2009)^k^	50	−8.099	36.484	−0.514	0.039	0.017	0.045	0.034	0.014	0.020		NS	NS	NS	*	NS	NS	NS	NS	NS	NS	NS	NS	NS	NS	*	*	*
Idete (2010)^k^	47	−8.099	36.484	−0.514	0.020	0.006	0.034	0.016	0.013	0.018	0.004		NS	NS	NS	NS	NS	NS	NS	NS	NS	NS	NS	NS	NS	NS	NS	*
Namawala (2008)^k^	40	−8.157	36.578	−0.504	0.034	0.021	0.052	0.039	0.027	0.030	0.010	0.009		NS	NS	NS	NS	*	NS	*	NS	*	*	NS	NS	*	NS	*
Namawala (2009)^k^	38	−8.157	36.578	−0.504	0.038	0.017	0.050	0.038	0.022	0.024	0.004	0.000	0.007		NS	NS	NS	NS	NS	NS	NS	NS	NS	NS	NS	NS	NS	*
Namawala (2010)^k^	48	−8.157	36.578	−0.504	0.020	0.019	0.040	0.028	0.013	0.030	0.016	0.004	0.016	0.013		*	NS	NS	NS	*	NS	NS	*	*	NS	NS	NS	*
Lupiro (2008)^k^	51	−8.385	36.678	−0.580	0.040	0.018	0.044	0.032	0.027	0.021	0.002	0.003	0.010	0.004	0.012		NS	NS	NS	NS	NS	NS	NS	NS	NS	NS	*	*
Lupiro (2009)^k^	43	−8.385	36.678	−0.580	0.030	0.021	0.046	0.036	0.014	0.020	0.007	−0.002	0.005	0.001	0.009	0.003		NS	NS	NS	NS	NS	NS	NS	NS	NS	NS	*
Lupiro (2010)^k^	42	−8.385	36.678	−0.580	0.030	0.026	0.029	0.021	0.004	0.027	0.014	0.003	0.021	0.007	0.004	0.014	0.005		NS	NS	NS	NS	NS	NS	NS	NS	NS	*
Sululu (2010)^k^	34	−8.003	36.837	−0.315	0.033	0.025	0.028	0.015	0.005	0.021	0.019	0.004	0.025	0.016	0.003	0.014	0.012	−0.007		NS	NS	NS	NS	NS	NS	NS	NS	*
Sawasawa (2010)^k^	51	−7.883	36.867	−0.241	0.036	0.016	0.027	0.014	0.009	0.023	0.003	0.001	0.011	0.010	0.015	0.005	0.005	0.005	0.007		NS	NS	NS	NS	NS	*	*	*
Chita (2010)^k^	45	−8.292	35.596	−0.989	0.035	0.022	0.042	0.025	0.016	0.020	0.004	0.001	0.002	0.002	0.002	−0.002	0.000	0.001	0.005	0.003		NS	NS	NS	NS	NS	*	*
Malinyi (2010)^k^	48	−8.943	36.139	−1.096	0.035	0.025	0.035	0.026	0.014	0.023	0.011	0.003	0.013	0.008	0.004	0.003	0.006	−0.002	0.000	0.006	−0.003		NS	NS	NS	*	*	*
Mahenge (2011)^k^	49	−8.897	36.717	−0.827	0.047	0.030	0.036	0.027	−0.001	0.019	0.013	0.007	0.020	0.015	0.020	0.018	0.010	0.004	0.004	0.003	0.011	0.014		NS	NS	*	*	*
Sagamanganga (2010)^k^	47	−8.067	36.800	−0.364	0.041	0.019	0.040	0.022	0.015	0.022	0.007	0.006	0.008	0.014	0.019	0.006	0.011	0.013	0.014	0.000	0.004	0.007	0.006		NS	*	*	*
Mkindo (2009)	38	−6.254	37.549	0.889	0.040	0.024	0.040	0.023	0.001	0.025	0.008	0.008	0.014	0.011	0.012	0.014	0.011	0.003	0.006	0.005	0.005	0.008	−0.001	−0.002		*	*	*
Mahuninga (2011)	48	−7.928	35.652	−0.778	0.007	0.007	0.040	0.028	0.015	0.028	0.015	−0.002	0.017	0.012	0.004	0.016	0.005	0.010	0.010	0.015	0.011	0.014	0.021	0.020	0.017		NS	*
Bahi (2011)	46	−5.950	35.312	0.100	0.003	0.017	0.043	0.035	0.017	0.042	0.024	0.007	0.019	0.018	0.003	0.027	0.010	0.008	0.012	0.023	0.015	0.017	0.028	0.028	0.021	−0.005		*
Insectary (2011)^k^	46	n/a	n/a	n/a	0.120	0.083	0.111	0.059	0.088	0.084	0.066	0.072	0.085	0.087	0.066	0.078	0.099	0.073	0.059	0.073	0.071	0.064	0.083	0.073	0.069	0.078	0.088	

Boxed F_ST_ and significance values are from temporal populations. The principal component (PC) reflects location explaining 87% and 84% of variation in latitude and longitude for *An. gambiae s.s.* (A) and *An. arabiensis* (B) collection sites respectively.

Significant and non-significant pairwise F_ST_ values are represented by an asterisk (*) and NS respectively. ^k^Samples from the Kilombero Valley.

**Figure 5:**
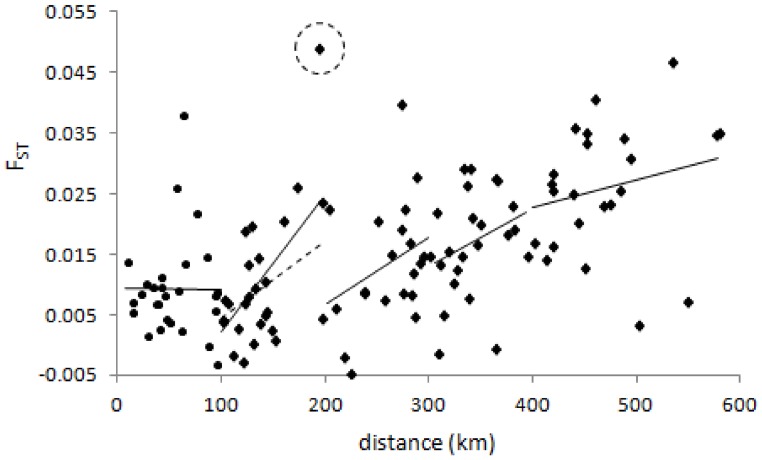
Variation in IBD slope with geographic scale in *An. arabiensis*. The isolation by distance slope is calculated separately for each 100 km class of pairwise distances (or 200 km class for 400–600 km, owing to fewer points). For the 101–200 km class the dashed line shows the slope if the outlying point (enclosed in dashed circle) is excluded.

**Figure 6:**
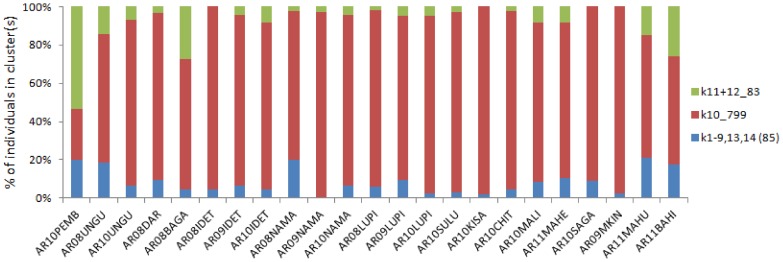
Individual-based BAPS spatially-conditioned clustering. Cluster identification numbers (k*n*) are shown, with number of individuals after the underscore.

The relationship between genetic differentiation and distance was also highly significant in *An. arabiensis* (Fig. 4C), and in contrast to *An. gambiae* s.s., the greater number and continuity of samples permitted closer inspection of the relationship. At the smallest spatial scale of distance comparisons (up to 100 km) there was no relationship between distance and differentiation, but in each broader scale category thereafter, the slope of the isolation by distance (IBD) relationship was quite consistent (Fig. 5). Therefore, apart from at a fine scale, distance was a reasonable predictor of genetic differentiation, concordant with gene flow limited by distance. Owing to this clear IBD relationship and near-complete lack of clustering using the BAPS group-level analysis, we also performed individual-level clustering analysis for *An. arabiensis* data (again using spatial information as a prior) to determine whether locations appeared especially differentiated. The resultant solution was dominated by one major cluster containing almost 83% of all individual samples, 10 very small clusters, which we pooled together to aid interpretation, and two similar clusters, which when pooled yielded a similar overall size to the 10 minor clusters. Though not conclusive, samples from the island of Pemba were less represented in the dominant cluster (Fig. 6), consistent with slightly greater differentiation than observed among the rest of the dataset.

### Evidence of population stability and *kdr* distributions

Owing to the fragmented nature of the *An. gambiae* s.s. distribution and known declines in frequency, we examined evidence for population instability via bottleneck tests. *An. arabiensis* populations which have not obviously declined in the same manner as *An. gambiae s.s.* have, were also tested, though consequently with a contrasting expectation. In both species, tests proved inconclusive, with results entirely dependent on the mutation model applied in simulations (Table S1A, B). We also attempted estimation of effective population size, *Ne*. All samples of *An. gambiae* s.s., with the exception of the insectary sample, exhibited an upper confidence interval of infinity, highlighting poor reliability of the estimates. Nevertheless it is interesting to note that, after the insectary, the Unguja and Bagamoyo samples were also the next most differentiated and exhibited the next lowest point *Ne* estimates, suggesting a possible role for isolation and genetic drift (Table S1C).

**Figure 7:**
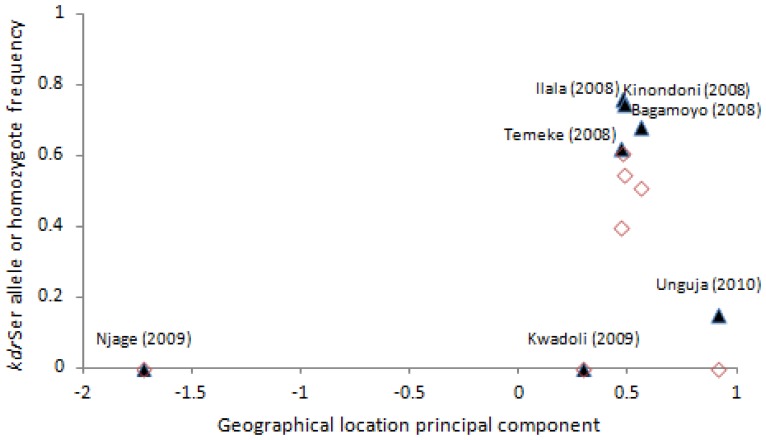
*Kdr* L1014S in *An. gambiae s.s.* Serine allele (filled triangles) and serine homozygote (open diamonds) frequencies are plotted against a principal component reflecting location (explaining 87% of variation in latitude and longitude).

*Kdr 1014F* was absent in all samples genotyped in this study. *Kdr* 1014S was almost entirely absent from *An. arabiensis*, with just a single heterozygote detected in Dar es Salaam (from 693 genotyped individuals), and a second heterozygote in one of the two *An. gambiae* s.s. x *An. arabiensis* hybrids found in the Dar es Salaam collections. In *An. gambiae* s.s., *kdr* 1014S was found at highly variable frequencies among sites, exceeding 70% in the three samples from Dar es Salaam and also Bagomoyo, but only 16% on the nearby island of Unguja, where only *kdr* heterozygotes were present. *Kdr* was absent from three additional sample sites, Njage, Kwadoli and Kilombero (Fig. 7). In all samples, *kdr* was in Hardy-Weinberg equilibrium. Although the relationship between *kdr* frequency and geographical location (measured using a principal component) was not significant (Spearman’s ρ = 0.46, p = 0.30), too few sample sites were available to confidently reject a hypothesis of distance-restricted *kdr* distribution. However, the extremely strong differentiation of Unguja from all mainland *An. gambiae* s.s. samples is more consistent with positive selection driving *kdr* frequencies following introduction of the allele by very occasional migration, rather than recurrent gene flow from the mainland.

## Discussion

Physical barriers, distance and environmental adaptation have all been implicated previously as causal factors reducing gene flow in *An. gambiae* s.l., but few studies have compared their roles in *An. gambiae* s.s. and *An. arabiensis*. Comparison of the population structure of each species in Tanzania was hindered by the relative rarity of *An. gambiae* s.s. in many areas. Indeed the more fragmented nature of the *An. gambiae s.s.* sampling scheme than in *An. arabiensis* in our study is a direct consequence of this. In spite of this limitation, both similarities and differences between the species were apparent. *An. gambiae* s.s. sample sites further apart were generally more differentiated than those nearby, with the notable exception of Unguja. This island population bore the hallmarks of isolation, specifically extremely high differentiation and much reduced genetic diversity. Furthermore, the absence of any clear signal of a population bottleneck suggests this may reflect a history of limited gene flow from the mainland. This extreme isolation of Unguja actually masked a strong correlation between distance and differentiation in *An. gambiae* s.s., though we suggest caution in interpretation of the underlying causation. IBD is expected when gene flow is limited by dispersal distance, leading to a stepping-stone population model whereby neighbouring locations are much more likely to exchange migrants [71]. IBD can also be indicative of migration-drift equilibrium [72], a state at which the link between differentiation and gene flow (c.f. differentiation and population history) becomes much closer [73], making patterns of differentiation easier to interpret for practical applications. However, with relatively limited and discontinuous sampling, and most inter-sample distances far exceeding the plausible dispersal range of an organism [74], such interpretation is problematic. This is the case for *An. gambiae* s.s. in our study, and thus we cannot conclude that distance is the causal factor in creating differentiation, or in limiting gene flow. Local factors influencing immigration or demography may also be important. In this context the relatively high differentiation of the coastal Bagamoyo sample, located approximately 60 km from those in Dar es Salaam, and the low but significant differentiation of the Temeke from Kinondoni and Ilala samples (7 km apart within Dar es Salaam) may be of note. Other East African studies (conducted in Kenya) have also reported relatively high genetic differentiation in *An. gambiae* s.s. populations sampled at small spatial scales, e.g. 50 km apart [75], [76].

*An. gambiae* s.s. has been systematically experiencing a remarkably rapid decline in the Kilombero Valley, with recent research showing this species comprises less than 1% of the *An. gambiae* complex in some villages where it used to be the dominant species [77]. Thus it might be expected that *An. gambiae* s.s. population in the valley would be experiencing a more isolated or patchy existence. However, we did not detect significant differentiation between samples within the valley. Though based on only a single pair of sites, this mirrors findings from another recent study in the area [43], which reported very low differentiation (average F_ST_ = 0.006).

In contrast to *An. gambiae* s.s., genetic diversity was invariant among *An. arabiensis* samples, most pairwise comparisons were not significant, and, with the exception of the insectary sample, cluster analysis failed to detect any significant partitions in the data. Even application of the potentially more sensitive individual-based spatial clustering method was inconclusive, though there was some suggestion of at least minor separation of the Pemba island sample. Does such weak structure reflect extensive gene flow, or even near-panmixia? The larger and more continuous sample set for *An. arabiensis* helps to answer this question. The relationship between genetic differentiation and distance was similarly strong for *An. gambiae* s.s. and *An. arabiensis*, but for the latter it was possible to examine the relationship at different spatial scales. This revealed a consistent IBD pattern for all but the finest scale of comparisons, consistent with migration-drift equilibrium [72], and in the absence of patterns of variation in genetic diversity, alternative explanations related to population spread and/or colonisation time are not supported [39], [78], [79]. Despite this support for a broad equilibrium scenario, which permits inference of gene flow from differentiation, direct conversion of F_ST_ to number of effective migrants per generation (*Nm*), though still commonplace in literature, is probably unwise for pairwise comparisons because high sampling error in F_ST_ [80] is compounded when converting to *Nm* [73]. However, as a rough indicator, average F_ST_ values for the distance classes translate to *Nm* estimates of between approximately 9 and 25, though literal interpretation as numbers of migrating individuals is unwise. In spite of widespread lack of significant differentiation, it is important to appreciate that *An. arabiensis* in Tanzania and the associated major islands are not panmictic, but rather conform to a stepping-stone model of semi-continuous population structure, with considerable gene flow limited primarily by distance. Such continuity suggests that vector control applied at a local scale could often be hampered by persistent re-colonisation, and potentially to a much greater extent than for Tanzanian *An. gambiae*. Again, in contrast to *An. gambiae* s.s. differentiation of Unguja was entirely unexceptional, and that of the more isolated island, Pemba, only slightly elevated above the majority of inter-site comparisons. This observation could reflect an impact of the sea as a partial barrier to gene flow, or more simply might arise from the location of Pemba at the end of a chain of sample sites; thus with more limited potential for immigration as a result of distance alone. Differentiation between the Zanzibar islands and mainland populations of *An. arabiensis* is dramatically less than that found in a previous study of differentiation among Madagascar, Reunion and Mauritius [34], perhaps reflecting the much closer proximity to the mainland of the Zanzibar islands.

Genetic differentiation among populations in the Kilombero Valley was generally very limited. Ng’habi et al [43] reported a very much higher level of genetic differentiation among *An. arabiensis* sample sites within the Kilombero Valley (F_ST_ = 0.066) which was attributed to the presence of a separate genetic cluster of *An. arabiensis*, which were in some sites but common in others, and highly divergent (F_ST_>0.1). It is possible a novel cryptic subgroup was discovered in the valley which gave rise to these results [43], but the cause of multiple deviations from Hardy-Weinberg equilibrium and extremely unusual patterns of linkage disequilibrium among markers does not appear to have been fully explored. Therefore, null alleles and scoring errors should first be discounted, especially (a) given the scoring problems we observed for some of the *An. gambiae* s.s. microsatellites when used to amplify *An. arabiensis*, and (b) the sensitivity of clustering to small numbers of highly differentiated markers [29].

There was slight, but significant genetic differentiation [Table 2B] among sequences of samples from *An. arabiensis* populations collected between 2008 and 2010 from Idete. Lupiro and Namawala had temporal populations collected within similar periods but did not show significant genetic differentiation to each other across time. Temporal sequences of samples from populations of *An. arabiensis* collected in 2008 and 2010 in Unguja Island were not significantly different from each other. Observation of absence of genetic differentiation among temporal populations of *An. arabiensis* have been reported by [23], [42], [81], although a study on temporal population structure of *An. gambiae* s.s. revealed significant genetic differentiation between successive monthly collections [75]. Lower levels of differentiation among *An. arabiensis* in our study may be explained by the larger effective population size evidenced in *An. arabiensis*, which maintains stable genotype frequencies across wet and dry seasons [23].

LLINs are widely used in most parts of Tanzania and especially in the Kilombero Valley. Behavioural differences in the two species, which render *An. gambiae* s.s. more amenable to control by LLINs, may have contributed to the decrease in *An. gambiae* s.s. numbers relative to its sister species *An. arabiensis* [8]–[10] as described above. Persistence of *An. gambiae* s.s. in more genetically isolated populations than found in *An. arabiensis* is consistent with the findings of the present study.

The pyrethroid and DDT-linked resistance mutation *kdr* 1014S [12], [82], [83] was found at extremely low frequency in *An. arabiensis*. This mirrors results from other studies in Tanzania [84], [85]. However, *kdr* 1014F was absent from all samples we tested, yet was recently found in samples of *An. arabiensis* from Dar es Salaam collected only three years later, (but not in *An. gambiae* s.s.) [85] at frequencies approaching those in *An. arabiensis* from Ethiopia and Sudan [86], [87]. *An. gambiae* s.s. populations from Dar es Salaam and Bagamoyo were found to have high frequencies of *kdr* 1014S (around 70%), with approaching 50% homozygotes. In Unguja Island 1014S was at much lower frequency, with no homozygotes. The other populations examined including one in the Kilombero Valley were wild type for *kdr*. The finding of 1014S at high frequency in this study is consistent with other recent reports in East Africa [14], [88], [89].

Absence of *kdr* from the Kwadoli and Njage (Kilombero valley) samples of *An. gambiae* s.s. highlights that, despite extensive use of LLINs for nearly 10 years, *kdr* has either failed to migrate into these populations or the populations have been subjected to reduced selection pressure compared to those on the coast. The link between amount of gene flow and transfer of insecticide resistance mutations in *Anopheles* is not well understood, though it seems that any quantitative connection may be weak. In West Africa, *kdr* 1014F has introgressed from S to M forms of *An. gambiae* (now termed *An. gambiae* s.s. and *An. coluzzii*), between which hybridisation is rare, and has subsequently risen to high frequency [90], [91]. Similarly in this study, differentiation of Unguja from Bagomoyo and Dar es Salaam is high enough to suggest a major barrier to recent gene flow, yet *kdr*, which we assume came from the mainland, is present at appreciable frequency. Preventing the spread of resistance alleles which are under sufficient selection pressure to almost ensure establishment in a population, which appears to be true for both of the *kdr* 1014 mutations, presents a difficult challenge.

## Supporting Information

Table S1 **Supplementary table showing results for bottleneck and effective population size tests.** BOTTLENECK (A, B) was run with three contrasting mutations with default settings for the TPM. A significant heterozygote excess (P<0.05, relative to equilibrium model expectations) provides evidence of a bottleneck, whereas a heterozygote deficit suggests population expansion. Effective population size estimates were calculated by LDNA using a minimum permissible allele frequency of 0.05. Confidence limits calculated by two methods (parametric and jackknifing are shown).
(DOCX)

Data S1 **Genotype and collection sites for all samples.**
(XLSX)

Data S2 **Allele count summaries for each collection site and locus (note that not all loci were included in final analysis - see main text).**
(XLSX)
