# Supplementary material for: Islands and Stepping-Stones: Comparative Population Structure of Anopheles gambiae sensu stricto and Anopheles arabiensis in Tanzania and Implications for the Spread of Insecticide Resistance
Source: PLoS One. 2014 Oct 29;9(10):e110910. doi: 10.1371/journal.pone.0110910 (PMC4212992; doi:10.1371/journal.pone.0110910)
Supplement: Table S1 — Supplementary table showing results for bottleneck and effective population size tests. BOTTLENECK (A, B) was run with three contrasting mutations with default settings for the TPM. A significant heterozygote excess (P<0.05, relative to equilibrium model expectations) provides evidence of a bottleneck, whereas a heterozygote deficit suggests population expansion. Effective population size estimates were calculated by LDNA using a minimum permissible allele frequency of 0.05. Confidence limits calculated by two methods (parametric and jackknifing are shown). (DOCX) [file pone.0110910.s001.docx]

| **Table S1**: **Supplementary table showing results for bottleneck and effective population size tests**. BOTTLENECK (A, B) was run with three contrasting mutations with default settings for the TPM. A significant heterozygote excess (P<0.05, relative to equilibrium model expectations) provides evidence of a bottleneck, whereas a heterozygote deficit suggests population expansion. Effective population size estimates were calculated by LDNA using a minimum permissible allele frequency of 0.05. Confidence limits calculated by two methods (parametric and jackknifing are shown). | | | | | | | | | | | | | | | | | | |
| --- | --- | --- | --- | --- | --- | --- | --- | --- | --- | --- | --- | --- | --- | --- | --- | --- | --- | --- |
| **A** | ***An gambiae s.s.*** |  |  |  |  |  |  |  |  |  |  |  |  |  |  |  |  |  |
|  | Bottleneck |  |  |  |  |  |  |  |  |  | **C** | **Ne** |  |  |  |  |  |  |
|  |  | **Wilcoxon test** |  | het xs |  |  |  | het def |  |  |  |  |  |  |  |  |  |  |
|  |  |  | IAM | TPM | SMM |  | IAM | TPM | SMM |  |  |  |  | Ne estimate | LCL_parametric | LCL_jacknife | UCL_parametric | UCL_jacknife |
|  |  | Unguja (2010) | 0.62 | 0.92 | 0.99 |  | 0.42 | 0.10 | 0.01 |  |  |  | Unguja (2010) | 136 | 25 | 14 | Infinite | Infinite |
|  |  | Ilala (2008) | 0.19 | 0.88 | 1.00 |  | 0.84 | 0.14 | 0.00 |  |  |  | Ilala (2008) | Infinite | 187 | 159 | Infinite | Infinite |
|  |  | Kinondoni (2008) | 0.00 | 0.54 | 1.00 |  | 1.00 | 0.50 | 0.00 |  |  |  | Kinondoni (2008) | Infinite | 100 | 83 | Infinite | Infinite |
|  |  | Temeke (2008) | 0.01 | 0.16 | 0.90 |  | 0.99 | 0.86 | 0.12 |  |  |  | Temeke (2008) | Infinite | 174 | 178 | Infinite | Infinite |
|  |  | Bagamoyo (2008) | 0.02 | 0.54 | 0.95 |  | 0.99 | 0.50 | 0.07 |  |  |  | Bagamoyo (2008) | 180 | 66 | 54 | Infinite | Infinite |
|  |  | Kwadoli (2009) | 0.00 | 0.42 | 0.99 |  | 1.00 | 0.62 | 0.01 |  |  |  | Kwadoli (2009) | Infinite | 123 | 94 | Infinite | Infinite |
|  |  | Kilombero (2009) | 0.01 | 0.86 | 1.00 |  | 0.99 | 0.16 | 0.00 |  |  |  | Kilombero (2009) | Infinite | 125 | 85 | Infinite | Infinite |
|  |  | Njage (2009) | 0.00 | 0.28 | 0.99 |  | 1.00 | 0.75 | 0.02 |  |  |  | Njage (2009) | 325 | 81 | 81 | Infinite | Infinite |
|  |  | Insectary (2011) | 0.50 | 0.86 | 0.99 |  | 0.54 | 0.16 | 0.02 |  |  |  | Insectary (2011) | 30 | 15 | 12 | 80 | 140 |
|  |  |  |  |  |  |  |  |  |  |  |  |  |  |  |  |  |  |  |
|  |  |  |  |  |  |  |  |  |  |  |  |  | Minimum allele freq permitted = 0.05 | | |  |  |  |
| **B** | ***An arabiensis*** |  |  |  |  |  |  |  |  |  |  |  |  |  |  |  |  |  |
|  | Bottleneck |  |  |  |  |  |  |  |  |  | **D** | **Ne** |  |  |  |  |  |  |
|  |  | **Wilcoxon test** |  | het xs |  |  |  | het def |  |  |  |  |  |  |  |  |  |  |
|  |  |  | IAM | TPM | SMM |  | IAM | TPM | SMM |  |  |  |  | Ne estimate | LCL_parametric | LCL_jacknife | UCL_parametric | UCL_jacknife |
|  |  | Pemba (2010) | 0.14844 | 0.76563 | 0.98828 |  | 0.94531 | 0.28906 | 0.01953 |  |  |  | Pemba (2010) | Infinite | 111.3 | 83.3 | Infinite | Infinite |
|  |  | Unguja (2008) | 0.02734 | 0.53125 | 0.97266 |  | 0.98047 | 0.53125 | 0.03906 |  |  |  | Unguja (2008) | Infinite | 133.2 | 74.4 | Infinite | Infinite |
|  |  | Unguja (2010) | 0.59375 | 0.96094 | 0.96094 |  | 0.46875 | 0.05469 | 0.05469 |  |  |  | Unguja (2010) | Infinite | 18.4 | 19.3 | Infinite | Infinite |
|  |  | Dar (2008) | 0.05469 | 0.8125 | 0.99219 |  | 0.96094 | 0.23438 | 0.01172 |  |  |  | Dar (2008) | 171.8 | 38.1 | 32.7 | Infinite | Infinite |
|  |  | Bagamoyo (2008) | 0.01172 | 0.40625 | 0.97266 |  | 0.99219 | 0.65625 | 0.03906 |  |  |  | Bagamoyo (2008) | 28.3 | 11.7 | 10.2 | 397.5 | Infinite |
|  |  | Idete (2008) | 0.14844 | 0.85156 | 0.99219 |  | 0.94531 | 0.1875 | 0.01172 |  |  |  | Idete (2008) | 166.3 | 41.1 | 37.2 | Infinite | Infinite |
|  |  | Idete (2009) | 0.05469 | 0.76563 | 0.76563 |  | 0.96094 | 0.28906 | 0.28906 |  |  |  | Idete (2009) | 245 | 56.8 | 44 | Infinite | Infinite |
|  |  | Idete (2010) | 0.00781 | 0.34375 | 0.97266 |  | 0.99609 | 0.71094 | 0.03906 |  |  |  | Idete (2010) | 254.8 | 59.7 | 50.5 | Infinite | Infinite |
|  |  | Namawala (2008) | 0.05469 | 0.65625 | 0.97266 |  | 0.96094 | 0.40625 | 0.03906 |  |  |  | Namawala (2008) | 55.1 | 25.7 | 22.3 | 350.2 | Infinite |
|  |  | Namawala (2009) | 0.02734 | 0.65625 | 0.99219 |  | 0.98047 | 0.40625 | 0.01172 |  |  |  | Namawala (2009) | 37.6 | 19.7 | 15 | 108.4 | 422.4 |
|  |  | Namawala (2010) | 0.03906 | 0.53125 | 0.97266 |  | 0.97266 | 0.53125 | 0.03906 |  |  |  | Namawala (2010) | 371.1 | 56.9 | 38.6 | Infinite | Infinite |
|  |  | Lupiro (2008) | 0.01953 | 0.59375 | 0.97266 |  | 0.98828 | 0.46875 | 0.03906 |  |  |  | Lupiro (2008) | 165.6 | 57.5 | 42.4 | Infinite | Infinite |
|  |  | Lupiro (2009) | 0.34375 | 0.71094 | 0.98828 |  | 0.71094 | 0.34375 | 0.01953 |  |  |  | Lupiro (2009) | Infinite | 95.3 | 57.1 | Infinite | Infinite |
|  |  | Lupiro (2010) | 0.05469 | 0.76563 | 0.98828 |  | 0.96094 | 0.28906 | 0.01953 |  |  |  | Lupiro (2010) | Infinite | 82.6 | 49.3 | Infinite | Infinite |
|  |  | Sululu (2010) | 0.14844 | 0.59375 | 0.98828 |  | 0.94531 | 0.46875 | 0.01953 |  |  |  | Sululu (2010) | 543.3 | 53.4 | 41.5 | Infinite | Infinite |
|  |  | Kisawasawa (2010) | 0.01172 | 0.59375 | 0.98047 |  | 0.99219 | 0.46875 | 0.02734 |  |  |  | Kisawasawa (2010) | 664.8 | 78.6 | 60.5 | Infinite | Infinite |
|  |  | Chita (2010) | 0.1875 | 0.8125 | 0.98047 |  | 0.85156 | 0.23438 | 0.02734 |  |  |  | Chita (2010) | Infinite | 108.7 | 63.8 | Infinite | Infinite |
|  |  | Malinyi (2010) | 0.1875 | 0.8125 | 0.98828 |  | 0.85156 | 0.23438 | 0.01953 |  |  |  | Malinyi (2010) | 162.5 | 45 | 34.7 | Infinite | Infinite |
|  |  | Mahenge (2011) | 0.02734 | 0.76563 | 0.98828 |  | 0.98047 | 0.28906 | 0.01953 |  |  |  | Mahenge (2011) | 582 | 64.4 | 60.4 | Infinite | Infinite |
|  |  | Sagamanganga (2010) | 0.00391 | 0.59375 | 0.98828 |  | 1 | 0.46875 | 0.01953 |  |  |  | Sagamanganga (2010) | 1455.4 | 74.8 | 58.3 | Infinite | Infinite |
|  |  | Mkindo (2009) | 0.01953 | 0.40625 | 0.99219 |  | 0.98828 | 0.65625 | 0.01172 |  |  |  | Mkindo (2009) | 30.2 | 17.6 | 15.1 | 62.5 | 87.2 |
|  |  | Mahuninga (2011) | 0.01172 | 0.59375 | 0.98047 |  | 0.99219 | 0.46875 | 0.02734 |  |  |  | Mahuninga (2011) | Infinite | 165.2 | 77 | Infinite | Infinite |
|  |  | Bahi (2011) | 0.23438 | 0.94531 | 0.98828 |  | 0.8125 | 0.14844 | 0.01953 |  |  |  | Bahi (2011) | Infinite | 96.5 | 89.1 | Infinite | Infinite |
|  |  | Insectary (2011) | 0.46875 | 0.97266 | 0.99219 |  | 0.59375 | 0.03906 | 0.01172 |  |  |  | Insectary (2011) | Infinite | 244.7 | 206.1 | Infinite | Infinite |
